# Incorporating social media into physician assistant education: opportunities to benefit patients

**DOI:** 10.5116/ijme.5cf0.43e1

**Published:** 2019-06-14

**Authors:** Jaideep S. Talwalkar, Elizabeth Roessler, Sumeet L. Banker, Ada M. Fenick

**Affiliations:** 1 Departments of Medicine and Pediatrics, Yale School of Medicine, New Haven, Connecticut, USA; 2 Department of Medicine, Yale School of Medicine, New Haven, Connecticut, USA; 3 Division of Child and Adolescent Health, Columbia University College of Physicians and Surgeons, New York, USA; 4 Department of Pediatrics, Yale School of Medicine, New Haven, Connecticut, USA

**Keywords:** Social media, physician assistant education, opportunities, patients, USA

## To the Editor

We read the recent paper by Wanner and colleagues^1^ with interest. The authors surveyed physician assistant (PA) students about their experiences with social media and concluded that educators might formally incorporate the use of social media into curricula designed for PA students to augment more traditional modalities like textbooks and lectures. We agree with the authors’ conclusions but would emphasize an additional benefit of weaving social media into PA student education – the benefit to their future patients.

Social media use is widespread among patients,^2^ including children and adolescents.^3^ The American Academy of Physician Assistants has advised PAs about standards of professionalism in their personal use of social media, largely centered around patient privacy.^4^ Other professional organizations such as the American College of Physicians^2^ and American Academy of Pediatrics^3^ emphasize the important role that clinicians should play in counseling about safe and appropriate social media use. For adults, this might include guidance about reputable sources of medical information,^2^ whereas discussions with younger patients might also include counseling about cyberbullying, sexting, and permanence of the digital footprint.^5^

We conducted a survey of PA students in their final semester at Yale University to gauge their own engagement with social media, and their attitudes and behaviors regarding the clinician’s role in counseling about social media. The electronic survey was sent to all 37 final semester PA students in the fall of 2016, 24 of whom (65%) responded. Similar to Wanner and colleagues,^1^ social media utilization among students was high, with all 24 (100%) respondents having engaged in social networking to stay personally connected with family, friends, and other trainees. Social media use for professional purposes was far less common with 7 (29%) having used social media to share medical information; 1 (4%) student was connected with faculty via social networks and 0 with patients or patients’ families. Despite high social media utilization, and agreement that “clinicians have a role in counseling” about social media use (17/24, 71%), only 3 (13%) felt comfortable doing this counseling, and 1 (4%) felt that she had received adequate training in the area.

Wanner and colleagues^1^ argue that formal inclusion of social media into PA student education was well received because the modality is familiar and engaging for the learners. In Wanner’s study, despite their familiarity with social media, many students felt it should be “an optional adjunct” in their education because of time demands and lack of reliability of online sources.^1^ The pervasiveness of social media justifies its use as an adjunct in medical education. However, student acceptance of social media as a formal part of the curriculum, rather than “an optional adjunct”, might have been improved if teaching *about* social media had accompanied teaching *with* social media.

Educators and clinicians must not forget that the pervasiveness of social media also affects our patients and their families in clinically meaningful ways. This point should be discussed with a generation of learners who, despite high levels of personal usage, still are quite reticent to discuss the positive and negative health and developmental effects of social media with their patients. In addition to engaging students with course content, we posit that the use of social media by educators would present an occasion to address learning opportunities identified by our survey and emphasized by professional organizations. Specifically, as social media takes hold in PA student education, discussions about the clinician’s role in discussing social media with patients and standards of professionalism would naturally follow.

### Conflict of Interest

The author declares that there are no conflicts of interest.
